# CoReCG: a comprehensive database of genes associated with colon-rectal cancer

**DOI:** 10.1093/database/baw059

**Published:** 2016-04-25

**Authors:** Rahul Agarwal, Binayak Kumar, Msk Jayadev, Dhwani Raghav, Ashutosh Singh

**Affiliations:** ^1^Department of Life Science, Shiv Nadar University, Greater Noida, India; ^2^Department of Health Research (Ministry of Health & Family Welfare), Division of Epidemiology and Communicable Diseases, Indian Council of Medical Research, Ansari Nagar, New Delhi, India

## Abstract

Cancer of large intestine is commonly referred as colorectal cancer, which is also the third most frequently prevailing neoplasm across the globe. Though, much of work is being carried out to understand the mechanism of carcinogenesis and advancement of this disease but, fewer studies has been performed to collate the scattered information of alterations in tumorigenic cells like genes, mutations, expression changes, epigenetic alteration or post translation modification, genetic heterogeneity. Earlier findings were mostly focused on understanding etiology of colorectal carcinogenesis but less emphasis were given for the comprehensive review of the existing findings of individual studies which can provide better diagnostics based on the suggested markers in discrete studies.

Colon Rectal Cancer Gene Database (CoReCG), contains 2056 colon-rectal cancer genes information involved in distinct colorectal cancer stages sourced from published literature with an effective knowledge based information retrieval system. Additionally, interactive web interface enriched with various browsing sections, augmented with advance search facility for querying the database is provided for user friendly browsing, online tools for sequence similarity searches and knowledge based schema ensures a researcher friendly information retrieval mechanism.

Colorectal cancer gene database (CoReCG) is expected to be a single point source for identification of colorectal cancer-related genes, thereby helping with the improvement of classification, diagnosis and treatment of human cancers.

**Database URL:** lms.snu.edu.in/corecg

## Background

Colorectal cancer (CRC) is a dreadful disease with global presence and is among the three most common malignancies reported worldwide with alarming numbers of incidence of 1 360 000 cases and 694 000 fatalities worldwide. The highest rate of incidence in both the sexes was reported from Australian continent followed by Western Europe and North America (1). Nonetheless, South East Asia region and particularly Indian subcontinent is witnessing much rise in incidence of CRC because of changing food habits, climate change or altered environmental conditions and sedentary (inactive) lifestyle (2). Approximately 64 000 cases (wherein 37 000 are men and 27 000 are women) and 49 000 deaths (wherein 28 000 are men and 21 000 are women) occurred due to colo-rectal carcinoma in India (3). CRC is treatable if detected in early stages for which diagnostics methods like fecal occult blood testing, flexible sigmoidoscopy, double-contrast barium enema X-ray and colonoscopy (Gold Standard) are used. However, the complexity and affordability issues together with high drug resistance rate has increased CRC treatment burden throughout the globe. For recurrence studies again Colonoscopy is Gold standard but expensive, invasive, frequently not readily available and occasionally has serious complications. Additionally, many are unwilling to undergo screening colonoscopy or computed tomography examinations of the colon (CT). Therefore, there is an urge for potential molecular biomarker which can precisely identify the CRC and should be affordable. To serve the purpose, carcinoembryonic antigen (CEA) a glycoprotein present in tissue as well as in serum is most commonly used as traditional biomarker (diagnostic and prognostic) but CEA levels does not provide sufficient sensitivity and reliability for the early detection of CRC, therefore CEA like single gene based biomarkers clinical utility is questionable (4). Therefore, comprehensive gene list could be instrumental for researchers to formulate a gene panel based diagnostic methods. Till date only two databases namely colon cancer gene variant databases (5) and CRCgene database (6) exists but, both of them have very small data set with 11 and 64 genes, respectively.

This study involves creation of highly curated comprehensive database of CoReCG, with 2056 genes referenced from 2486 published evidences (articles) which contain all factual colon-rectal cancer-related genes information with an effective knowledge based information retrieval system. Moreover, a comparison of CoReCG database has also been made with existing cancer gene databases like catalog of somatic mutations in cancer (COSMIC) (7), Cancer Genes (8), network of cancer genes (NCG) (9), CanGeneBase (10), Cancer3D (11), renal cancer gene database (RCDB) (Renal) (12), genes-to-systems breast cancer (G2SBC) (Breast Cancer) (13), cervical cancer gene database (CCDB) (Cervical Cancer) (14) and few more. CoReCG provides colon-rectal carcinoma genes with features like gene, mRNA, protein information, structures, chromosomal location, architecture, chromosome number, functions, homology, families, coding sequences (CDS) information, genomic coordinates, single nucleotide polymorphism (SNP) information, chromosomal map, domain information, pathway information, ontology information, drug information, etc. Hyperlink to other web resources like Genbank (15), HGNC (16), Uniprot (17), Ensembl (18), University of California, Santa Cruz (UCSC) (19), dbSNP (20), protein data bank (PDB) (21), Kyoto encyclopedia of genes and genomes (KEGG) (22), String (23), conserved domain database (CDD) (24), human protein reference database (HPRD) (25), consensus coding sequence (CCDS) (26, 27) and online Mendelian inheritance in man (OMIM) (28) make CoReCG well equipped for advance searching. Further, the database provide various browsing sections, with searching options for querying the database and online tools for sequence similarity searches like basic local alignment search tool (BLAST) (29), CoReCG also has genome browser to display gene information graphically with the coordinates (30).

The comprehensive study of CoReCG genes reveals some significant observations like maximum number of genes (1211) are involved in phosphorylation process followed by acetylation (365 genes) and glycosylation (163 genes) as its post-translation modification function. Also, substantiate from the biological process study that most of the genes (322) are engaged in cell communication and/or 661 genes in signal transduction processes. CoReCG also provides information on progression of disease by browsing CoReCG through stage giving information on sample source and stage specific features of the genes.

## Construction and content

**Figure 1.**
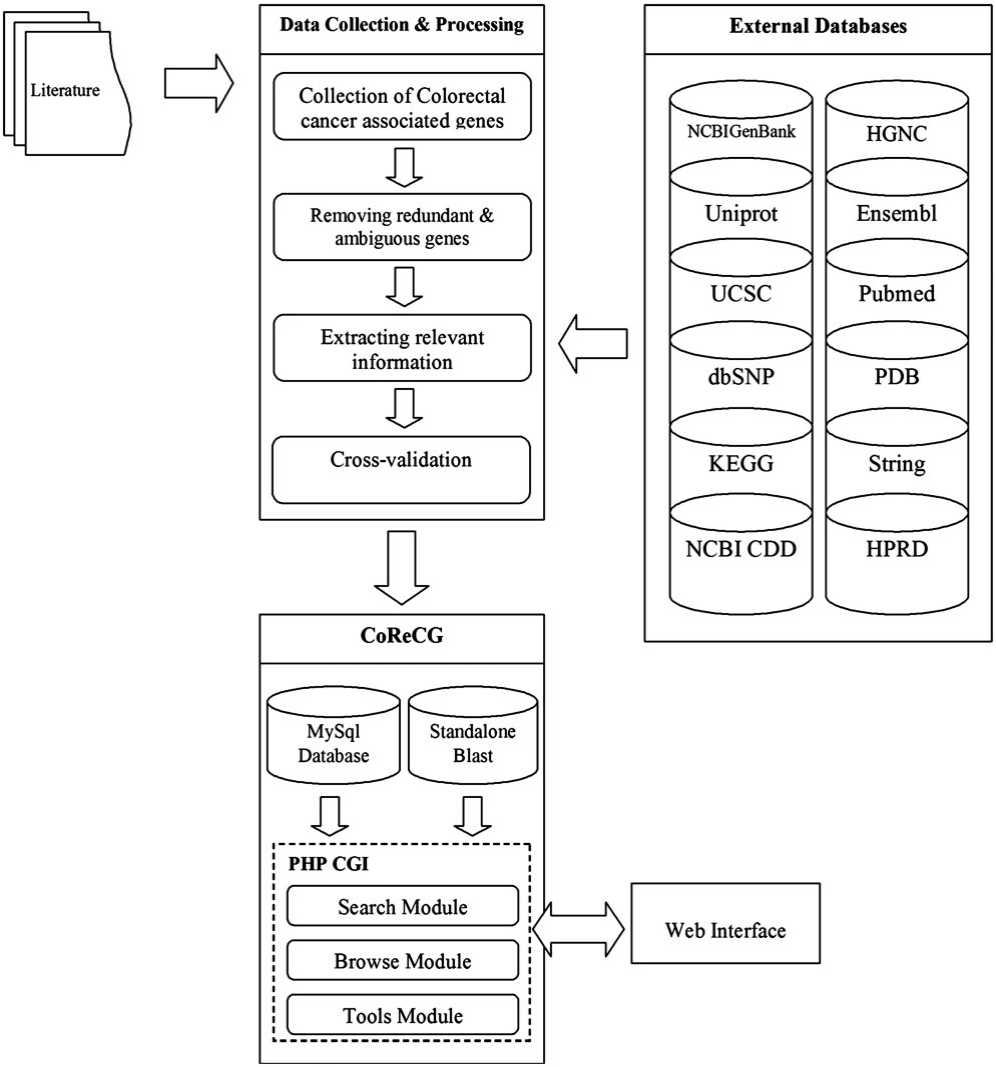
The schema of CoReCG database. Figure explains the data collection steps used in CoReCG resourced from literature and various databases. It also showing methods for retrieval of information and databases linked to CoReCG.

The primary motivation of CoReCG is to collect and maintain a high quality CRC genes database, which serve as a major resource for CRC-associated genes and will definitely fill the gap of a well annotated composite database for CRC responsive genes. The schema of the CoReCG is represented in Figure 1.

## Data acquisition

**Figure 2.**
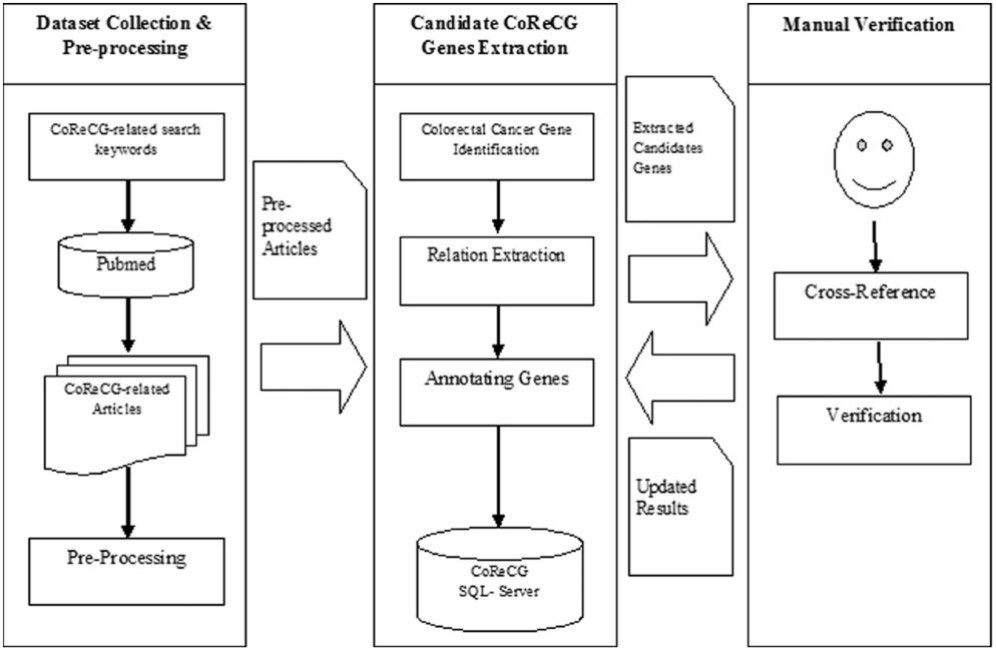
The flowchart of CoReCG data collection. The figure shows the steps involved in collecting data for CoReCG which includes, preprocessing of data using related keywords against pubmed to fetch relevant articles, followed by the extraction of information from literature and annotation and submitting information in CoReCG, the whole process were manually curated and verified from cross references and updating in CoReCG.

Initial entries describing the relationships between genes and CRC were collected manually. Extensive search strategies were exercised for creating the repository, array of keywords were used like; ‘colorectal cancer genes’, ‘colon cancer genes’, ‘rectum cancer genes’, ‘bowel cancer genes’ and a few more to collect literatures containing information regarding genes associated with the CRC. The search results were limited to those published before November 2015. Additionally, genes from existing cancer databases were also searched in literature for documenting relationship with colon cancer. The research articles were exploited to extract important information like mechanism (such as methylation, gene amplification, mutation, altered expression and polymorphism), experiment and special comment on the gene related information and specific remark were also obtained and properly reported in the database. Further, to make it more informative and clinician friendly, significance of the gene alteration with CRC sample sources that is the location from which the sample was collected by the respective authors to conduct the study, experimental methods which were involved in the study to validate the involvement of genes in CRC, fold change in case of over-expression and down-regulation and some other information like staging, sample type, sample sizes and other important characteristics have been incorporated. All the aforementioned features have been introduced in the evidence information table which can also be retrieved via pubmed ID (PMID) key. Figure 2 depicts the flowchart for the CoReCG data collection. CoReCG contains information collected from 2486 published research articles. Table 1 shows the statistics of the CoReCG evidence data.

**Table 1.** Literature statistics of CoReCG

Publication year	Number of papers
1980–2000	**64**
2001–2005	**201**
2006–2010	**624**
2011–**2015**	**1597**

## Post-processing and annotating genes

In post-processing, first the gene identifiers (i.e. Entrez gene IDs) were extracted manually. Thereafter, the entries which cannot be reliably mapped to entrez gene, such as ambiguous gene names or retired Gene IDs without further traceable information were excluded. Subsequently supplementary annotations were performed to produce complete detailed information. The annotation has made the CoReCG a single point reference for all the colon-rectal cancer-associated genes, which may nullify or greatly reduce the time of user in maneuvering at multiple databases. Additionally, Gbrowse v2.54 has been added which provides a quick and easy-to-use visual display of genomic data like motif, domain, CDS and gene. It places annotation tracks beneath genome coordinate positions, allowing rapid visual correlation of different types of information.

## Database design and implementation

The CoReCG is implemented in MySQL and is designed to make it easily accessible via the internet and permit users to search for genes according to specific criteria. The online release of the database functions in all major browsers and is compatible with mostly all operating systems. The web interface of CoReCG database is written in PHP and JavaScript under Apache web server running on a Linux system.

## Database content

**Figure 3.**
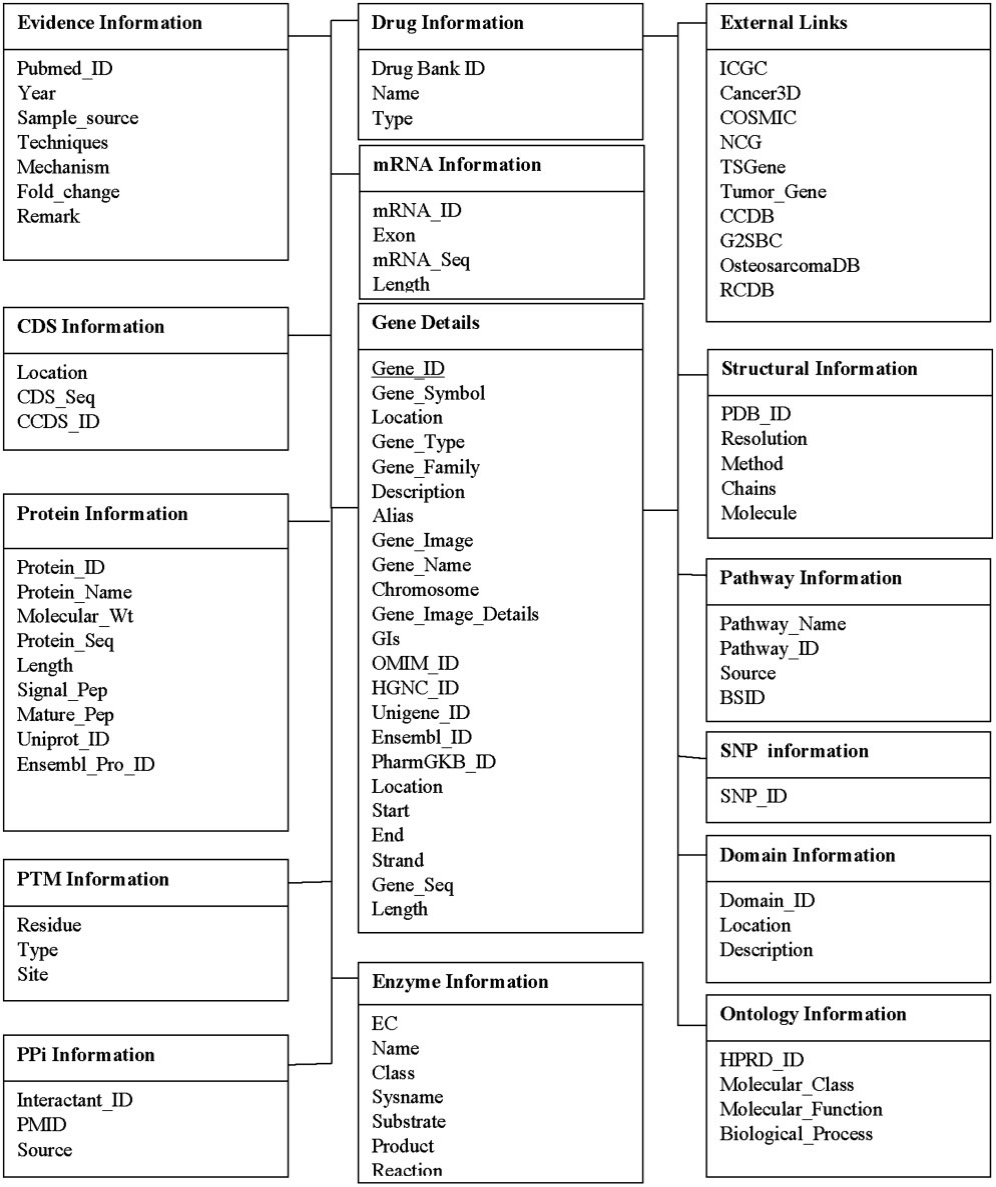
Database structure of CoReCG. The detailed MySQL database structure which consists of 14 tables.

The MySQL database consists of total 15 inter–intra connected tables, dealing with the information on gene characteristics, location, product information, citing information (as shown in Figure 3).

(i) The evidence information table gives information about the published source and cited information for each gene listed on the database. (ii) gene information, (iii) mRNA information, (iv) CDS information, (v) protein information, (vi) post-translational information, (vii) enzyme information, (viii) PPi information, (ix) pathway information, (x) structural information, (xi) domain information, (xii) ontology information, (xiii) SNP information, (xiv) drug information and (xv) external links.

## Utility and discussion

The database web portal consists of several user-friendly features to facilitate access to the data which provide users with additional tools for analysis. These include:

Search utility: Basic and Advance search.

Browse utility

Tools utility

**Figure 4.**
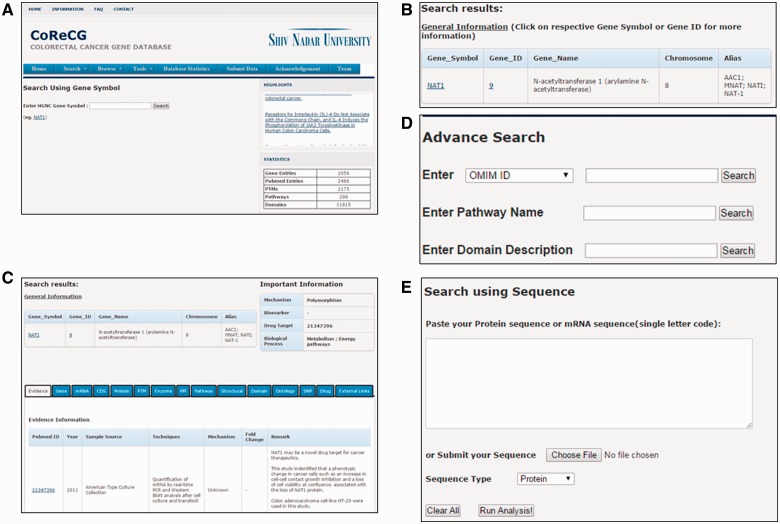
Web interface of CoReCG. (**A**) Search CoReCG using a keyword (Gene Symbol). (**B**) Query result obtained after keyword search. (**C**) Detailed information obtained after selecting a gene id. (**D**) Advance Search page of CoReCG. (**E**) Search CoReCG using a sequence.

CoReCG provides two types of search interface:

**(i) Keyword search interface**—provides an interface for searching the CoReCG database with several keywords such as gene symbol, gene ID and chromosome. For example, if a keyword ‘NAT1’ is provided (Figure 4A), the query result will be displayed in a tabular format, with the features of gene symbol, gene ID, gene name, chromosome and alias (Figure 4B). Further accessing the link of gene ID, the detailed information for gene NAT1 will be shown (Figure 4C). The gene information, accounted gene symbol, full name, summary, reference information, ontology information and pathway information. The gene, mRNA, CDS, protein sequence, genomic location and some useful external links are also presented. All output fields are also indicated in Figure 4C. Along with the basic keyword search CoReCG is also having an advanced search option which is more powerful than the basic search. In advance search, we can search CoReCG by using various other database IDs like OMIM, Unigene, Ensembl Gene, PharmGKB, CCDS, Uniprot, Ensembl Protein, HPRD and HGNC ID, also with the pathway name and Domain name/description. Both pathway name and domain description is having autosearch option for facilitating user in searching. Figure 4D is showing the advance search option of CoReCG.

**(ii) Sequence search interface**—Sequence search option provides an interface for searching the CoReCG database with protein or nucleotide sequence (Figure 4E). For example, if a sequence ‘>NAT1

 MDIEAYL ERI GYKKSRNKLDLETLTDILQH QIRA V P F ENLNIHCGD AMDLGLEAIFDQVVR RNRGGWC LQ V N HLLY WALTTIGFETTMLGGYVYST PAKKY STG MI H LLLQVTIDGRN’ is given, the corresponding result will be shown in tabular format, comprising the respective features of gene ID, the gene IDs possessing similar sequence as the input sequence will be listed.

## Browse and tools utility

CoReCG web interface provides two advanced features, i.e. browse and tools utility. In browse utility, information of CoReCG database could be browsed by any of mentioned terms, i.e. biomarker/drug target, gene information, stage, pathway, ontology and mechanism. Additionally, while browsing using gene information one can also sort through gene ID, gene symbol, gene name, alias and chromosome. The order of genes in the table can be sorted by multiple columns in a sequential manner by clicking the upward and downward arrows in the header row. CoReCG provides a tool utility where user can perform following analysis; (i) Blast—user can use an online Blast interface to input an interested sequence in fasta format and search against all nucleotide and protein sequences in CoReCG database. (ii) CoReCG GBrowse—user can visualize the genomic coordinates of CoReCG genes. CoReCG GBrowse displays information like gene coordinates, CDS coordinates, open reading frame and Motifs/Domain.

**Figure 5.**
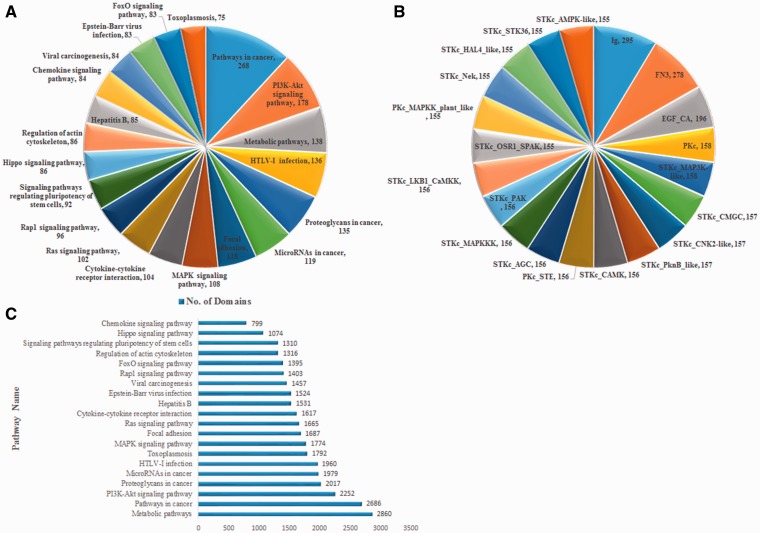
(A) Major pathways shared by the genes. (**B**) Major domains shared by the genes. (**C**) Number of domains present in various major pathways.

CoReCG provides a comprehensive collection of manually curated CRC-associated genes. The main aim of the CoReCG is to help the CRC research community by providing a platform where all the detailed information of genes involved in CRC is available. The information in CoReCG database are stored in such a manner that user can easily find whether a gene or protein is associated with CRC or not. Analysis of CoReCG revealed that database genes are involved in 288 pathways obtained from KEGG pathways. Also, 11 815 different domains obtained from National Center for Biotechnology Information (NCBI) CDD database are present in these genes. Major pathways shared by the genes are shown in Figure 5A and the major domains shared by the genes are shown in Figure 5B. Figure 5C is showing the relationship between the domain and biological pathways.

Gene ontology terms are widely used to characterize protein function and to elucidate trends in protein datasets. All CRC-associated genes were classified according to the molecular function of each protein and biological process in which it is involved. Assignment of 2056 genes to various molecular functions revealed top five categories: Transcription factor activity, transcription regulator activity, protein serine/threonine kinase activity, receptor activity and catalytic activity thereby suggesting importance of these gene products in development of CRC cells. Biological processes found to be enriched in CoReCG are (i) cell communication and/or signal transduction: 47.8%, (ii) regulation of nucleobase, nucleoside, nucleotide and nucleic acid metabolism: 10.7% and (iii) energy pathways: 8.5%. There are 2175 post-translation modifications associated with CoReCG genes in which (i) phosphorylation—occurs in 1211 genes, i.e. 58.9%, (ii) acetylation—occurs in 365 genes, i.e. 17.7% and (iii) glycosylation—occurs in 163 genes, i.e. 7.9%. These data suggests that phosphorylation is an important post-translation modification involved in the CoReCG genes that involve in the progression or may be in regulation of CRC.

Further, enzyme information is extracted to identify the enzymes that are associated with CRC. Enzymes are widely used as a drug target for various disease treatments including cancer. Among 2056 CRC-associated genes, enzyme information available only for 600 genes which were found to be distributed as transferases (252 gene product), hydrolases (206 gene products), oxidoreductases (83 gene product) and Isomerases, Lyases and Ligases share 9, 12 and 38 gene products, respectively.

CoReCG genes manual annotation also provides understanding to study the mechanism for causation of disease, majorly 5 mechanisms were found to be involved in: (i) over-expression—1153 genes (56%), (ii) down-regulation—418 genes (20.3%), (iii) polymorphism—213 genes (10.3%), (iv) methylation—199 genes (9.6%) and (v) mutation—183 genes (8.9%). This suggests that differential gene expression plays a critical role in cancer progression as around 76% of genes are either up-regulated or down-regulated.

Single repository having validated information about the genes involved in CRC is missing. Currently only two database exists for CRC one is colon cancer gene variant databases [4] with 11 genes and CRCgene databbase [5] with 64 genes. CRCgene databbase has documented results of a meta-analysis to identify the polymorphism reported in different genes in CRC. Additionally, it lacks user-friendly search engine. Both studies primarily focused on variations and polymorphisms in the given set of genes.

We compared CoReCG with the available cancer gene databases to find the common and distinct genes. The comparison is shown in Table 2.

**Table 2.** CoReCG compared with other available cancer related databases

	Database name (Pubmed ID)	No. of genes	Common genes in database and CoReCG	Unique genes in database	Unique genes in CoReCG
**1**	**COSMIC (**7**)**	**522**	**214**	**308**	**1842**
**2**	**Cancer genes (**8**)**	**3387**	**979**	**2408**	**1077**
**3**	**NCG 5.0 (**9**)**	**1571**	**450**	**1121**	**1606**
**4**	**CanGeneBase (**10**)**	**140**	**73**	**67**	**1983**
**5**	**Cancer3D (**11**)**	**255**	**99**	**156**	**1957**
**6**	**RCDB (**12**)**	**237**	**128**	**109**	**1928**
**7**	**G2SBC (**13**)**	**2278**	**743**	**1535**	**1313**
**8**	**CCDB (**14**)**	**538**	**271**	**267**	**1785**
**9**	**CTdatabase (**31**)**	**283**	**22**	**261**	**2034**
**10**	**TSGene 2.0 (**32**)**	**1018**	**451**	**567**	**1605**
**11**	**Tumor-associated gene (**33**)**	**799**	**370**	**429**	**1686**
**12**	**ChimerDB (**34**)**	**431**	**106**	**325**	**1950**
**13**	**Bushmanlab (**35**)**	**2125**	**778**	**1347**	**1278**
**14**	**Osteosarcoma database (**36**)**	**911**	**489**	**422**	**1567**
**15**	**DDPC (**37**)**	**700**	**396**	**304**	**1660**
**16**	**OncoDBHCC (**38**)**	**611**	**270**	**341**	**1786**
**17**	**HNOC (**39**)**	**451**	**262**	**189**	**1794**
**18**	**Tumor gene family of databases (**40**)**	**467**	**99**	**368**	**1957**
**19**	**PC-GDB (**41**)**	**119**	**76**	**43**	**1980**

CoReCG on the other hand is a composite knowledgebase which provides a comprehensive collection of manually curated CRC-related genes which can work as gene signatures, gathered from decades of molecular profiling studies on CRC. CoReCG database could be utilized for ranking the genes for further in-depth molecular diagnostics studies.

In future, we will enrich the database with CRC related miRNA with the synergistic network. The group will update CoReCG genes database annually.

## Conclusions

The database is available at lms.snu.edu.in/corecg, it is a comprehensive database of CRC-associated genes and it may serve as the reference database for genes showing abrupt behavior in CRC. It may provide lead for potential biomarker and for cross referencing results of colon cancer transcriptomics experiments.

## Availability and requirements

The database is freely available at http;//www.lms.snu.edu.in/CoReCG/. For structure visualization, user should have Java runtime enabled in their web browser.
